# Iron Oxide Nanoparticles as Promising Antibacterial Agents of New Generation

**DOI:** 10.3390/nano14151311

**Published:** 2024-08-03

**Authors:** Tian-Guang Zhang, Chao-Yu Miao

**Affiliations:** Department of Pharmacology, Second Military Medical University/Naval Medical University, Shanghai 200433, China

**Keywords:** metal oxide nanoparticles, iron oxide, antimicrobial resistance, antibacterial agent

## Abstract

Antimicrobial resistance (AMR) is growing into a major public health crisis worldwide. The reducing alternatives to conventional agents starve for novel antimicrobial agents. Due to their unique magnetic properties and excellent biocompatibility, iron oxide nanoparticles (IONPs) are the most preferable nanomaterials in biomedicine, including antibacterial therapy, primarily through reactive oxygen species (ROS) production. IONP characteristics, including their size, shape, surface charge, and superparamagnetism, influence their biodistribution and antibacterial activity. External magnetic fields, foreign metal doping, and surface, size, and shape modification improve the antibacterial effect of IONPs. Despite a few disadvantages, IONPs are expected to be promising antibacterial agents of a new generation.

## 1. Introduction

AMR is growing into a major public health crisis worldwide and has been prioritized by the World Health Organization (WHO) as one of the top 10 global public health threats to humanity [1,2]. Without proactive solutions, it is estimated that global deaths due to AMR could reach up to 10 million annually by 2050 [3]. The increasing AMR and the reducing alternatives to conventional agents prompt the development of novel strategies to treat microbial diseases. Nanoparticles have received significant attention in medical sciences over the past decade due to their unique physicochemical properties [4,5]. Metal-based nanoparticles are among the most promising novel antimicrobial agents because of their strong antimicrobial activity [6,7,8,9]. Not only are some of the mechanisms of action they use different from those of traditional agents, but they also target multiple biomolecules, delaying the development of resistant strains [6,9].

IONPs are the most preferable nanomaterials in biomedicine, primarily due to their relative safety and magnetic properties [10]. The major forms of IONPs are magnetite (Fe_3_O_4_) and its oxidized forms, maghemite (γ-Fe_2_O_3_) and hematite (α-Fe_2_O_3_). Superparamagnetic iron oxide nanoparticles (Fe_3_O_4_ and γ-Fe_2_O_3_) can be used in an increasing number of biomedical applications, including but not limited to targeted drug delivery, magnetic resonance imaging (MIR), and magnetic hyperthermia, depending on their advantages such as an ability to be guided to a selected location by an external magnetic field, magnetic resonance imaging, and the generation of heat in an alternating magnetic field [11]. In 1957, the potential of IONPs for the heating of tumor tissue under an alternating magnetic field was examined for the first time [12], and magnetic hyperthermia was invented in 1993 [13]. In 1960, the concept of magnetic targeting was proposed and magnetic NPs are successfully accumulated in the body by magnetic field [14]. In 1996, the first phase I clinical trial of magnetic drug targeting using IONPs was conducted on patients bearing cancer [15]. Also occurring in 1996, the United States Food and Drug Administration (FDA) approved the first contrast agent based on IONPs [16]. Though magnetic hyperthermia was in the development stage, the FDA approved the first iron-replacement agent for the treatment of iron deficiency anemia in 1992 [17].

Iron is an essential element for most microorganisms. Bacteria also have an obligate requirement for iron to support their growth and survival. However, iron has double-edged sword effects on bacteria. High levels of iron are detrimental to bacteria. Bacteria have evolved a variety of mechanisms to maintain iron homeostasis, which not only allow them to survive under iron-restricted conditions but also protect them from iron-induced free radical damage under iron-rich conditions [18,19]. IONPs can result in cell iron overload. Thus, IONPs have also exhibited great antimicrobial activity against microorganisms, including bacteria, fungi, and viruses [8].

In this review, focusing on the antibacterial properties of IONPs, we introduce their potential mechanisms of action against pathogenic bacteria, their structure, the main factors influencing their antibacterial activity and biodistribution, and their biocompatibility. Additionally, we point out the disadvantages of IONPs and propose approaches for enhancing their antibacterial effect.

## 2. Antibacterial Potential Mechanisms of IONPs

**Figure 1:**
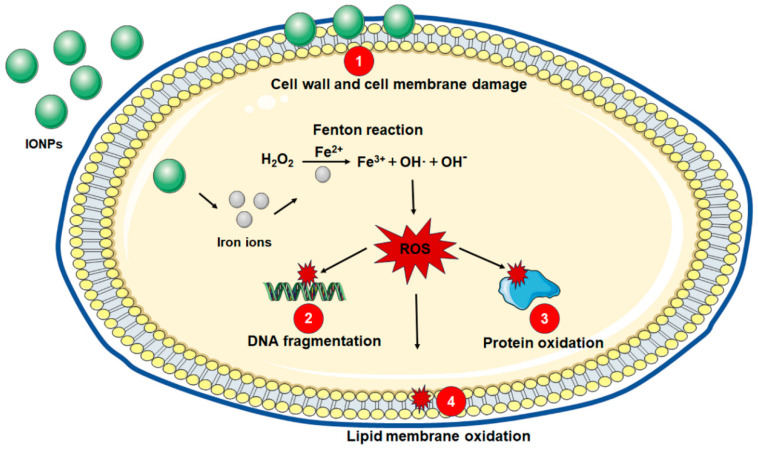
Antibacterial potential mechanisms of IONPs. (1) Cell wall and cell membrane damage via electrostatic and van der Waals interaction. (2) DNA fragmentation, (3) protein oxidation, and (4) lipid membrane oxidation are caused by ROS.

Two main mechanisms are involved in the antibacterial action of IONPs, including damage to the cell wall and cell membrane through an electrostatic and van der Waals interaction and oxidative stress by ROS formation (Figure 1).

Bacterial membranes contain highly electronegative chemical groups that serve as the binding sites of metal cations [20]. Metal oxides promote adhesion to the surfaces of negatively charged bacteria, primarily due to their positive charge [21]. IONPs can adhere to the cell wall and cell membrane of bacteria by the electrostatic and van der Waals interaction [22]. Then, IONPs form clusters on the bacterial surface and cause cell wall and cell membrane disruption, leading to leakage of bacterial content [23].

The most important mechanism of antibacterial effects of IONPs is the induction of oxidative stress by the production of ROS such as superoxide anion, hydroxyl radical and nonradical hydrogen peroxide through Fenton or Fenton-like reactions [24]. After internalization in bacteria, iron ions are released from IONPs and react with hydrogen peroxide to produce ROS, which may directly damage DNA, lipids, and protein, resulting in the accumulation of oxidative injury and thus bacterial death [25].

The Fenton reaction occurs when hydrogen peroxide is converted into a hydroxyl free radical via a Fe^2+^-catalyzed process, and Fe^2+^ is an accelerator for this reaction. Fenton oxidants (such as hydroxyl radicals) are generated in the cells of *Escherichia coli* exposed to bactericidal antibiotics [26], suggesting that the Fenton reaction occurs in bacteria. IONPs can act as a source of ferrous ions and serve as the role of catalysts [27]. A recent study evaluated the antibacterial activity of superparamagnetic iron oxide nanoparticles (SPIONs), ferrous and ferric ions against *Staphylococcus aureus* and *Escherichia coli* [28]. Under aerobic conditions, ferrous ions showed the strongest inhibitory effect against the growth of both stains. In fact, the antimicrobial effect of ferric ions depends on their conversion to ferrous ions by bacteria [29]. As the SPIONs showed less ROS production and antibacterial activity than ferrous, it could be concluded that similar to ferrous ions, the main mechanism of antibacterial effects for SPIONs was ROS generation.

## 3. Structure of IONPs

Due to their high surface energies from the great surface-to-volume ratio, bare IONPs have a high tendency to aggregate into large particles to reduce the energy of the surface, resulting in a loss of magnetism. Moreover, due to the high chemical activity of their surface, bare IONPs are easily oxidized, causing a loss of magnetism. Therefore, coating IONPs is essential to make them stable and biocompatible. Organic and inorganic materials can function as a coating for IONPs. The core–shell structure is typical for IONPs. In this structure, the iron oxide core was encapsulated in an inorganic or organic coating, which renders the particle stable.

Organic compounds have good biodegradability and biocompatibility and can provide functional groups such as carboxyl, hydroxyl, thiol, and amino groups. These groups not only possess strong binding affinity towards IONPs but also enable linkage with active bio-substances such as drugs and antibodies for bio-applications. Organic compounds include small-molecule organic and polymeric-coating materials. Oleic acid and trisodium citrate can stabilize nanoparticles mainly by steric repulsive forces to balance the magnetic and van der Waals attractive forces [30], thereby producing highly uniform IONPs. 2,3-Dimercaptosuccinic acid (DMSA), with two carboxyl groups and two sulfhydryl groups, is another typical organic surface-coating compound of IONPs [31]. The DMSA binds to the IONP surface through its carboxyl groups and strengthens the nanocrystal stability. The remaining carboxylic acid groups supply the surface with a negative charge, which makes IONPs hydrophilic and is easy for further conjugation of other bio-substances. In contrast to most small molecules, polymers bind to nanoparticles by multiple groups, contributing to stronger steric repulsive forces. Due to the excellent colloidal stability of IONPs, polymeric-coating materials are the most used in IONPs. Natural and synthetic polymers such as polyethylene glycol (PEG) and chitosan are used to coat IONPs. PEG is a hydrophilic, uncharged polymer whose biocompatibility has been recognized by the FDA [32,33]. The only disadvantage of PEG is that it is not easily biodegraded under biological conditions [34]. Chitosan is a positively charged, hydrophilic polymer with good biocompatibility and biodegradability [35,36]. The positive charge can drive the chitosan-based nanoparticles to the negatively charged cell membrane. Owing to the cell adhesion and positive charge, chitosan is a frequent choice for drug delivery [37]. Furthermore, chitosan has a broad antimicrobial spectrum and is susceptible to a variety of Gram-negative and Gram-positive bacteria [35].

Inorganic compounds such as SiO_2_, gold (Au), and silver(Ag) are able to increase the antioxidant properties of IONPs [38]. Silica is one of the extensively used coating materials due to its hydrophilicity, biocompatibility, and ability to prevent nanoparticle aggregation [39]. The negatively charged surface of silica, derived from the deprotonation of silanol groups, stabilizes the IONPs coated in silica by electrostatic repulsive forces. In addition, the abundant silanol groups of the silica can be easily activated and offer perfect anchorages for various functional groups [40]. Coating IONPs with melt provides a relatively inert layer with increased biocompatible and stability [41,42]. Similar to the gold coating, the electron transfer between silver and IONPs creates a positively charged silver coating, allowing the conjugation of different antibiotics to the silver-decorated IONPs by electrostatic interaction [43,44].

## 4. Main Factors Influencing the Antibacterial Activity of IONPs

The unique physicochemical properties of IONPs have a profound effect on antibacterial activity. The size, shape, and surface charge of IONPs are some of the main factors determining the antibacterial activity.

Size effects on the adsorption of α-Fe_2_O_3_ nanoparticles were studied in *Escherichia coli*. Adsorption of large α-Fe_2_O_3_ nanoparticles (76 and 96 nm) on *Escherichia coli* cells, which took 30–40 min, reached equilibrium faster than small nanoparticles, which took approximately 60–90 min [45]. However, the size of nanoparticles is inversely proportional to their bacterial activity. Small Fe_2_O_3_ nanoparticles tend to be more toxic than large nanoparticles for *Pseudomonas putida* because small nanoparticles have a relatively larger surface area to volume ratio than large nanoparticles, resulting in a great increase in the production of ROS [46,47]. Another study also showed that small-size Zero-valent iron nanoparticles with an average diameter (~35 nm) could rapidly inactivate *Escherichia coli* [48].

Various types of nanostructures of IONPs have been prepared, such as nanosphere, nanorod, nanowire, nanocrystal, and so on [49]. Sphere-shaped iron oxide nanoparticles and rod-shaped iron oxide nanoparticles were found to show good antibacterial activity [50]. Rod-shaped IONPs are shown to be more toxic than sphere-shaped IONPs [51]. This is because, unlike spherical nanoparticles, nonspherical particles such as rod-shaped nanoparticles can increase the propensity of nanoparticle–cell wall contact and potential penetration through the cell wall [52].

Positively charged IONPs have been shown to have higher antimicrobial activity than negatively charged ones, as the negative charge of the bacterial cell wall more easily electrostatically attracts the positively charged IONPs [53]. Due to the increased negative net charge of the outer membrane as a result of their composition, Gram-negative bacteria interact more with positively charged IONPs than Gram-positive bacteria, which lack a cell envelope structure [6]. A study explored the antibacterial properties of both negatively charged IONPs and positively charged IONPs against Gram-positive *Bacillus subtilis* and Gram-negative *Escherichia coli* [54]. The experiments showed higher antimicrobial activity of positively charged IONPs than negatively charged IONPs, and positively charged IONPs are more toxic to *Escherichia coli* than *Bacillus subtilis.* In addition, positively charged and neutral IONPs showed higher toxicity on *Streptococcus mutans* biofilms in comparison with their negatively charged counterparts, highlighting the effect of the surface charge of IONPs on antibiofilm activity [55].

## 5. Main Factors Influencing the Biodistribution of IONPs

**Figure 2:**
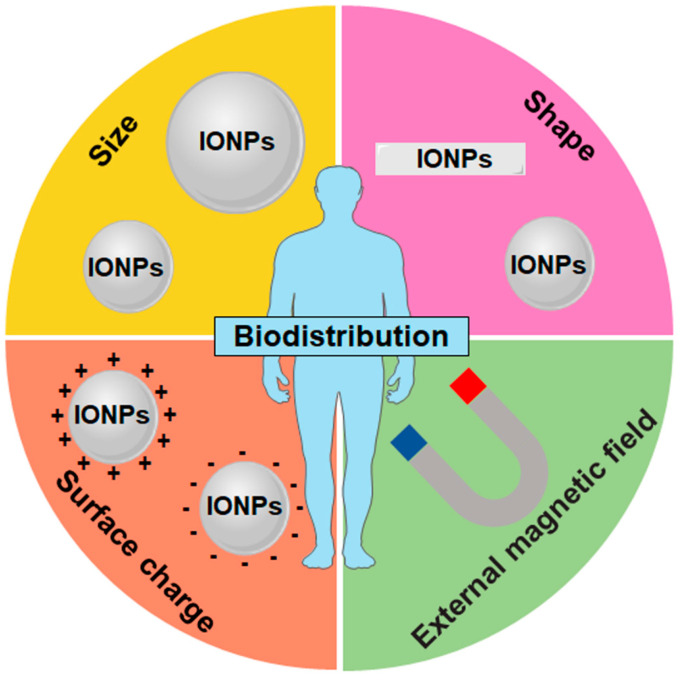
Main factors influencing the biodistribution of IONPs. Surface charge, shape, size, and superparamagnetism influencing the biodistribution of IONPs.

In addition to the ability to be directed to the desired tissues and organs by an external magnetic field, the size, shape, and surface charge of IONPs also influence their biodistribution in different organs (Figure 2 and Table 1). Intravenously injected IONPs are mainly accumulated and cleared in the liver and spleen by mononuclear phagocytic system (MPS) such as hepatic Kupffer cells and splenic macrophages [56,57]. For spherical IONPs, large-sized nanoparticles (>100 nm) accumulate readily within the liver and spleen, whereas small-sized nanoparticles (<15 nm) are eliminated by the kidneys [58].

Size is one of the primary factors determining the biodistribution of nanoparticles; however, shape is also as important. Discoidal particles favor their interaction with vessel–wall due to tumbling and oscillatory effects as well as more binding and contact points with the cell wall, compared with spherical particles [52]. For nanoparticles in the range of 20–150 nm, discoidal particles have been shown to accumulate more easily in the lung, liver, and spleen than spherical particles [52]. However, nanoparticles with a large length-to-width aspect ratio may increase their in vivo blood circulation time, owing to the tendency to align with blood flow and slower macrophage internalization [59,60]. For example, nanotube-shaped magnetic iron oxide nanoworms prolonged blood circulation times over their spherical counterparts [61].

The surface of IONPs is rapidly covered by blood plasma proteins in vivo after injection. The surface charge affects protein adsorption on the IONP surface and their MPS elimination. Adsorption of plasma proteins on the IONPs increases the size andthus MPS elimination [62]. Positively charged IONPs have a higher affinity to plasma proteins and show faster blood clearance in comparison with negatively charged IONPs [63,64]. Nanoparticles with a neutral or negative surface charge have been shown to have a longer circulation time due to a reduction in the adsorption of serum proteins [65]. Furthermore, negatively charged IONPs show a much lower affinity with cell membranes and less internalization due to the repulsive interactions with cell membranes [66]. Positively charged particles, such as positive charge IONPs, are more easily bound to macrophages in the lung, liver, and spleen, whereas neutral and negatively charged nanoparticles are less likely to accumulate in these organs [52,67].

**Table 1:** Antibacterial properties of IONPs.

Type	Size (nm)	Shape	Surface Charge	MIC (µg/mL)	MBC (µg/mL)	Bacteria	Ref.
Chitosan-Fe_3_O_4_	10–20	Spherical	Positive	NR NR	NR NR	*E. coli* *B. subtilis*	[54]
PVA-Fe_2_O_3_/Fe_3_O_4_	9 ± 4	Spherical	Negative	NR	NR	*S. aureus*	[68]
Amine-Fe_3_O_4_	6–15	Spherical	Negative	NR NR	NR NR	*S. aureus* *E. coli*	[69]
Arg-Fe_3_O_4_	6–15	Spherical	Negative	NR	NR	*S. aureus*	
APTMS -Fe_3_O_4_	6–15	Spherical	Positive	125 125	NR NR	*S. aureus* *E. coli*	
Oleic acid-Fe_3_O_4_	6–15	Spherical	Negative	31 63	NR NR	*S. aureus* *E. coli*	
Fe_3_O_4_ @PEG-Ag	20–25	Spherical	NR	16 16	32 32	*E. coli* *S. aureus*	[70]
Fe_3_O_4_@Ag	60 ± 20	Spherical	NR	≥70 ≥60 ≥70	NR NR NR	*E. coli* *S. epidermidis* *B. subtilis*	[71]
CES-Fe_3_O_4_	13.8 ± 2.1	Spherical	Negative	NR NR	NR NR	*S. aureus* *S epidermidis*	[72]
APTES-Fe_3_O_4_	17.8 ± 2.6	Spherical	Positive	NR NR	NR NR	*S. aureus* *S epidermidis*	
Fe_3_O_4_	13.7 ± 2.1	Spherical	Positive	NR NR	NR NR	*S. aureus* *S epidermidis*	
TEPSA-Fe_3_O_4_	12.1 ± 0.5	Spherical	Negative	NR	NR		[55]
TPED-Fe_3_O_4_	11.4 ± 0.4	Spherical	Positive	NR	NR	*S. mutans Biofilm*	
Fe_3_O_4_	10.1 ± 0.6	Spherical	Positive	NR	NR		
Fe_3_O_4_@APTES	17	Spherical	Positive	NR	NR	*B. subtilis biofilm*	[73]
Fe_2_O_3_	35.16 ± 1.47	Spherical	NR	NR NR NR NR	65 ± 1.5 120 ± 2.3 80 ± 1.5 78 ± 1.4	*E. coli* *P. aeruginosa* *S. aureus* *B. subtilis*	[74]
α-Fe_2_O_3_	20–30	Spherical	Positive	>150 >150	NR NR	*V. cholerae* *E. coli*	[75]
CEL/γ-Fe_2_O_3_/Ag	15–20	NR	NR	512 1024	1024 1024	*S. aureus* *E. coli*	[76]
FeO	20–80	Rod	Negative	NR	NR	*E. coli* *K. pneumoniae* *S. aureus*	[77]

MIC: minimum inhibitory concentration; MBC: minimum bactericidal concentration; NR: not reported; Ref.: reference; PVA: polyvinyl alcohol; APTMS: amino-propyl trimethoxy silane; CES: carboxyethylsilanetriol; APTES: 3-aminopropyltriethoxysilane; TEPSA: 3-(triethoxysilyl) propylsuccinic anhydride; TPED: N-[3-(trimethoxysilyl)propyl] ethylenediamine; APTES: 3-aminopropyltriethoxy silane; CEL: cellulose.

## 6. Biocompatibility of IONPs

IONPs present low toxicity in the human body. After internalization in eukaryotic cells, the IONPs are presumably degraded into iron ions in the lysosomes, and then these iron ions are released to the cytoplasm [46]. These iron ions are either incorporated in hemoglobin in red blood cells or eliminated from the body through the kidneys [78]. Due to their broad safety margin, several IONPs have been approved by the FDA for clinical application as imaging contrast agents and iron-replacement agents, as mentioned above. Among several other metal oxide nanoparticles, IONPs display an acceptable safety profile and no or low cytotoxicity [79]. Cytotoxicity tests for human glia, breast cancer, and normal cell lines demonstrated that Fe_3_O_4_ nanoparticles showed almost nontoxicity at doses of <10 μg/mL [80]. No clinically significant side effects have been reported for dextran-coated IONPs (e.g., Ferumoxytol) by intravenous injection and silica-coated IONPs (e.g., Ferumoxsil) by oral administration according to the standard pharmacological tests [81,82].

## 7. Disadvantages of IONPs

For metal oxide nanoparticles, the antimicrobial action of IONPs is relatively weak [22]. The inhibitory action of Fe_2_O_3_ nanoparticles on both Gram-negative *Escherichia coli* and *Pseudomonas aeruginosa* and Gram-positive *Staphylococcus aureus* and *Bacillus subtilis* are less effective than ZnO or CuO nanoparticles [74]. There was even a study that showed that IONPs could promote the growth of *Pseudomonas aeruginosa* grown in iron-deficient media due to an exogenous iron source for the bacteria [83]. In addition, the non-specificity of the antimicrobial action of IONPs on pathogens and symbiotic microbes can be detrimental to the host. IONPs may inhibit symbiotic microbes within the gastrointestinal tract, which provide many benefits to the host, such as the promotion of host immunity.

Biosafety is important when applying IONPs in biomedical applications. IONPs have bacteriostatic and bactericidal activities, whereas excess iron can be lethal to not only bacterial cells but also eukaryotic cells. Most nanotoxicities from IONPs derive from the aforementioned production of ROS from the nanoparticle surface or iron ions release [33]. In spite of low cytotoxicity, some IONPs have also been reported to cause toxic effects related to the nanoparticle size, administered dose, and age or preexistent pathological state of the study animals [84].

## 8. Approaches for Increasing the Antibacterial Effect of IONPs

**Figure 3:**
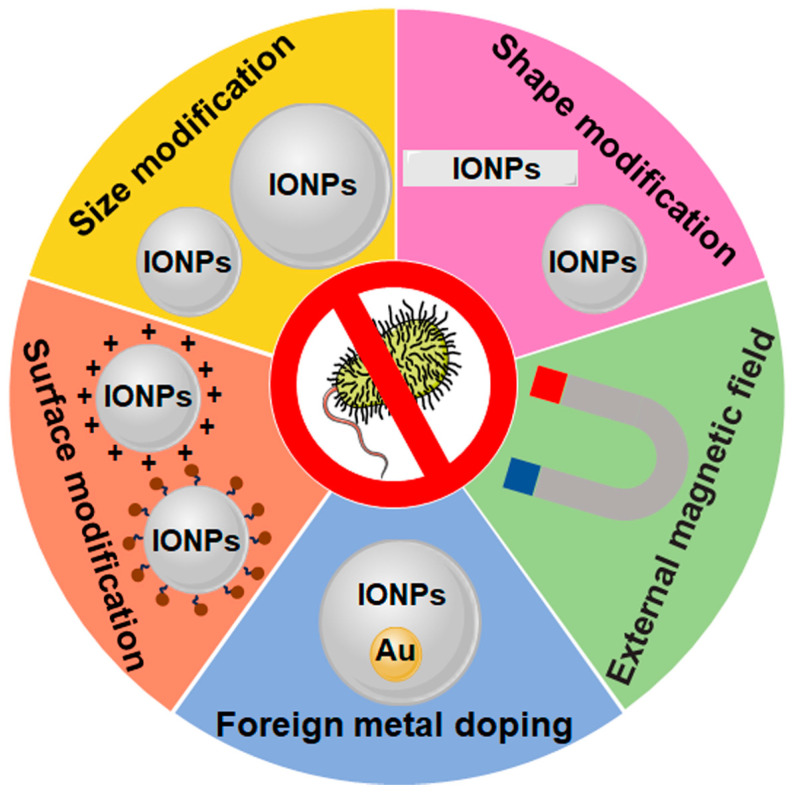
Approaches for increasing the antibacterial effect of IONPs. Surface, shape, size modification, and external magnetic field application improve both the antibacterial activity and biodistribution of IONPs, besides the improvement of antibacterial activity by foreign metal doping. The brown sphere represents bio-substances such as antibiotics or β-glucan.

The antimicrobial effect of IONPs can be improved by enhancing their antibacterial activity through external magnetic field application, foreign metal doping, and surface, shape, and size modification, as well as by influencing their biodistribution related to their size, shape, surface charge, and superparamagnetism (Figure 3 and Table 2).

SPIONs possess superparamagnetism, allowing them to undergo magnetization only under a magnetic field. It is notable that SPIONs have the capability to penetrate into bacteria and biofilms and produce localized heat under an external magnetic field. The use of an alternating magnetic field can dramatically improve the antibacterial activity of mesoporous hollow Fe_3_O_4_ nanoparticles (MHFPs) against *Escherichia coli* and *Staphylococcus aureus* and disperse bacterial biofilms due to the elevated local temperature and vibration damage [85]. Without an external magnetic field, 1000 μg mL^−1^ MHFPs induced a 22.70% and 23.54% reduction in CFU mL^−1^ for *Escherichia coli* and *Staphylococcus aureus* separately. However, the final counts of CFU mL^−1^ showed that there were 99.15% and 79.88% reductions in *Escherichia coli* and *Staphylococcus aureus*, individually with 1000 μg mL^−1^ MHFPs after external magnetic field treatment for 25 min.

Doping foreign metals into IONPs exhibited higher antimicrobial activity than IONPs [89,90,91]. Doping is the controlled insertion of a foreign element into the unoccupied crystal lattice to alter the characteristics of nanoparticles [94]. Doped IONPs alter the size and magnetic properties, facilitating interactions with bacteria and increased the charge density of magnetic nanoparticles, promoting ROS production [95]. The size of Fe_3_O_4_/ZnO nanoparticles decreased with the Ni dopant concentration, promoting antibacterial activity [90]. The average size of Fe_3_O_4_/ZnO was 36 nm, and the size decreased to 33 nm and 28 nm after the doping of 3% and 5% Ni individually. The zone of inhibition value of the Fe_3_O_4_/ZnO nanoparticles for both *Staphylococcus aureus* and *Klebsiella pneumoniae* was 6 mm individually. Furthermore, the 3% Ni-doped Fe_3_O_4_/ZnO nanocomposite increased the zone of inhibition value to 10–11 mm, and higher inhibitory zone values were found to be 11–12 mm for *Klebsiella pneumoniae* and 13–14 mm for *Staphylococcus aureus* for 5% Ni. Another study showed that the antibacterial activity of Ag-doped α-Fe_2_O_3_ nanoparticles was higher than that of α-Fe_2_O_3_ nanoparticles [89]. For *Staphylococcus aureus and Bacillus*, the zone of inhibition values were about 10 mm for α-Fe_2_O_3_ nanoparticles, and the corresponding values were about 14 mm for 5% Ag-doped nanoparticles. The main challenge of using doped IONPs is the choice of doping material. Incorporating hypotoxic metals such as Ag [96] or Au [97] are beneficial to the biocompatibility of doped IONPs.

Surface modification is also an important way to improve the antibacterial properties of IONPs. Hydrophobic negatively charged oleic acid-coated IONPs (OA-IONPs) show stronger antibacterial effects on Gram-positive *Staphylococcus aureus* and Gram-negative *Escherichia coli* compared to the unmodified IONPs [69]. Furthermore, 100 μg mL^−1^ OA-IONPs showed a 61% and 54% reduction in the count of CFU mL^−1^ for *Staphylococcus aureus* and *Escherichia coli*, respectively, whereas the unmodified IONPs had no effect on the growth of these bacteria at the same concentration. Positively charged chitosan-coated IONPs promote interactions with bacteria at the interfaces, resulting in enhancements of ROS production and antimicrobial activity [54]. The viability of *Bacillus subtilis* and *Escherichia coli* was reduced by approximately 30% by 50 μM unmodified IONPs, whereas the chitosan-coated IONPs had a significant effect on bacterial viability, and their viability was reduced by approximately 70% in the presence of chitosan-coated IONPs at the same concentration. Coating IONPs with trained immunity agonist β-glucan enhanced the antibacterial action of the IONPs against *Escherichia coli*, macrophage engulfment of pathogens, and the prevention and treatment of sepsis and secondary infections by inducing trained immunity [92]. Mucilage-coated IONPs, loaded with ciprofloxacin (Fe_3_O_4_@QSM-CIP), possess stronger antibacterial activity against standard strains of both Gram-positive and Gram-negative bacteria [93]. Specifically, the zone of inhibition values of Fe_3_O_4_ nanoparticles for *Bacillus cereus*, *Escherichia coli*, *Salmonella typhimurium*, and *Staphylococcus aureus* were 6.4, 7.2, 7.6, and 6.4 mm, whereas the corresponding values of Fe_3_O_4_@QSM-CIP extended to 19.9, 24.8, 29.7, 35.8 mm individually. Additionally, size modification and shape modification are alternative approaches to antibacterial activity improvement for IONPs. Applications of relatively small-sized or nonspherical IONPs are preferred due to their stronger antibacterial activity, as mentioned above.

Owing to the biodistribution of IONPs being influenced by their size, shape, surface charge, and superparamagnetism, as previously mentioned, the antibacterial effect of IONPs on some specific tissues and organs can also be improved by governing their biodistribution behavior through modulating the size, shape, and surface charge, besides directing them to the desired site of action using an external magnetic field.

## 9. Conclusions

Bacterial resistance evolves faster than the creation of new antibacterial agents. The uniqueness of magnetic IONPs brings benefits to their use in antibacterial applications. Among metal oxide nanoparticles, the antibacterial activity of IONPs is relatively weak, whereas IONPs have better biocompatibility [98]. Furthermore, external magnetic fields, foreign metal doping, and surface, size, and shape modification can enhance the antibacterial effect of IONPs. In view of a balance between antimicrobial action and biocompatibility, IONPs are expected to be potential antimicrobial agents of a new generation.

## Figures and Tables

**Table 2:** Approaches including external magnetic field, foreign metal doping, and surface modification for increasing the antibacterial effect of IONPs.

Method	Nanoparticle Type	Bacteria	Ref.
External magnetic field	Mesoporous hollow Fe_3_O_4_	*E. coli* *S. aureus*	[85]
	Hydroxyapatite-Fe_3_O_4_	*S. aureus* *E. coli*	[86]
	SPION-encapsulating polymersome	*S. epidermidis biofilms*	[87]
	Fe_3_O_4_@Ag@Hydroxyapatite	*S. aureus biofilms*	[88]
Foreign metal doping	Ag-doped α-Fe_2_O_3_	*S. aureus* *Bacillus* *Klebsiella* *E. col*	[89]
	Ni-doped Fe_3_O_4_/ZnO	*S. aureus* *K. pneumoniae*	[90]
	Au-doped Fe_3_O_4_	*A. baumannii* *S. enterica* *S. aureus* *M. luteus*	[91]
Surface modification	Oleic acid-Fe_3_O_4_	*S. aureus* *E. coli*	[69]
	Chitosan-Fe_3_O_4_	*B. subtilis* *E. coli*	[54]
	β-glucan-Fe_2_O_3_	*E. coli.*	[92]
	Fe_3_O_4_@QSM-CIP	*B. cereus* *E. coli* *S. typhimurium* *S. aureus*	[93]

QSM: quince seed mucilage; CIP: ciprofloxacin; Ref.: reference.

## Data Availability

Not applicable.

## References

[1] Salam M.A., Al-Amin M.Y., Salam M.T., Pawar J.S., Akhter N., Rabaan A.A., Alqumber M.A.A. (2023). Antimicrobial Resistance: A Growing Serious Threat for Global Public Health. Healthcare.

[2] World Health Organization 10 Global Health Issues to Track in 2021. https://www.who.int/news-room/spotlight/10-global-health-issues-to-track-in-2021.

[3] O’Neill J. Tackling Drug-Resistant Infections Globally: Final Report and Recommendations. http://amrreview.org/sites/default/files/160525_Final%20paper_with%20cover.pdf.

[4] Yusuf A., Almotairy A.R.Z., Henidi H., Alshehri O.Y., Aldughaim M.S. (2023). Nanoparticles as drug delivery systems: A review of the implication of nanoparticles’ physicochemical properties on responses in biological systems. Polymers.

[5] Mabrouk M., Das D.B., Salem Z.A., Beherei H.H. (2021). Nanomaterials for biomedical applications: Production, characterisations, recent trends and difficulties. Molecules.

[6] Slavin Y.N., Asnis J., Häfeli U.O., Bach H. (2017). Metal nanoparticles: Understanding the mechanisms behind antibacterial activity. J. Nanobiotechnol..

[7] Alavi M., Kamarasu P., McClements D.J., Moore M.D. (2022). Metal and metal oxide-based antiviral nanoparticles: Properties, mechanisms of action, and applications. Adv. Colloid Interface Sci..

[8] Sharmin S., Rahaman M.M., Sarkar C., Atolani O., Islam M.T., Adeyemi O.S. (2021). Nanoparticles as antimicrobial and antiviral agents: A literature-based perspective study. Heliyon.

[9] Sánchez-López E., Gomes D., Esteruelas G., Bonilla L., Lopez-Machado A.L., Galindo R., Cano A., Espina M., Ettcheto M., Camins A. (2020). Metal-based nanoparticles as antimicrobial agents: An overview. Nanomaterials.

[10] Malhotra N., Lee J.S., Liman R.A.D., Ruallo J.M.S., Villaflores O.B., Ger T.R., Hsiao C.D. (2020). Potential toxicity of iron oxide magnetic nanoparticles: A review. Molecules.

[11] Dulińska-Litewka J., Łazarczyk A., Hałubiec P., Szafrański O., Karnas K., Karewicz A. (2019). Superparamagnetic iron oxide nanoparticles-current and prospective medical applications. Materials.

[12] Gilchrist R.K., Medal R., Shorey W.D., Hanselman R.C., Parrott J.C., Taylor C.B. (1957). Selective inductive heating of lymph nodes. Ann. Surg..

[13] Jordan A., Wust P., Fähling H., John W., Hinz A., Felix R. (1993). Inductive heating of ferrimagnetic particles and magnetic fluids: Physical evaluation of their potential for hyperthermia. Int. J. Hyperth..

[14] Freeman M.W., Arrott A., Watson J.H.L. (1960). Magnetism in medicine. J. Appl. Phys..

[15] Lübbe A.S., Bergemann C., Riess H., Schriever F., Reichardt P., Possinger K., Matthias M., Dörken B., Herrmann F., Gürtler R. (1996). Clinical experiences with magnetic drug targeting: A phase I study with 4’-epidoxorubicin in 14 patients with advanced solid tumors. Cancer Res..

[16] Hamm B., Staks T., Taupitz M., Maibauer R., Speidel A., Huppertz A., Frenzel T., Lawaczeck R., Wolf K.J., Lange L. (1994). Contrast-enhanced MR imaging of liver and spleen: First experience in humans with a new superparamagnetic iron oxide. J. Magn. Reson. Imaging.

[17] Shah A., Dobrovolskaia M.A. (2018). Immunological effects of iron oxide nanoparticles and iron-based complex drug formulations: Therapeutic benefits, toxicity, mechanistic insights, and translational considerations. Nanomedicine.

[18] Andrews S.C., Robinson A.K., Rodríguez-Quiñones F. (2003). Bacterial iron homeostasis. FEMS Microbiol. Rev..

[19] Wandersman C., Delepelaire P. (2004). Bacterial iron sources: From siderophores to hemophores. Annu. Rev. Microbiol..

[20] Zhang Y.M., Rock C.O. (2008). Membrane lipid homeostasis in bacteria. Nat. Rev. Microbiol..

[21] Li B., Logan B.E. (2004). Bacterial adhesion to glass and metal-oxide surfaces. Cool. Surf. B.

[22] Shkodenko L., Kassirov I., Koshel E. (2020). Metal oxide nanoparticles against bacterial biofilms: Perspectives and limitations. Microorganisms.

[23] Li Y., Yang D., Wang S., Li C., Xue B., Yang L., Shen Z., Jin M., Wang J., Qiu Z. (2018). The detailed bactericidal process of ferric oxide nanoparticles on E. coli. Molecules.

[24] Tian Q., Xue F., Wang Y., Cheng Y., An L., Yang S., Chen X., Huang G. (2021). Recent advances in enhanced chemodynamic therapy strategies. Nano Today.

[25] Zúñiga-Miranda J., Guerra J., Mueller A., Mayorga-Ramos A., Carrera-Pacheco S.E., Barba-Ostria C., Heredia-Moya J., Guamán L.P. (2023). Iron oxide nanoparticles: Green synthesis and their antimicrobial activity. Nanomaterials.

[26] Belenky P., Ye J.D., Porter C.B., Cohen N.R., Lobritz M.A., Ferrante T., Jain S., Korry B.J., Schwarz E.G., Walker G.C. (2015). Bactericidal antibiotics induce toxic metabolic perturbations that lead to cellular damage. Cell Rep..

[27] Zha S., Cheng Y., Gao Y., Chen Z., Megharaj M., Naidu R. (2014). Nanoscale zero-valent iron as a catalyst for heterogeneous Fenton oxidation of amoxicillin. Chem. Eng. J..

[28] Gholami A., Mohammadi F., Ghasemi Y., Omidifar N., Ebrahiminezhad A. (2020). Antibacterial activity of SPIONs versus ferrous and ferric ions under aerobic and anaerobic conditions: A preliminary mechanism study. IET Nanobiotechnol..

[29] Sun H.Q., Lu X.M., Gao P.J. (2011). The Exploration of the Antibacterial Mechanism of FE(3+) against Bacteria. Braz. J. Microbiol..

[30] Soares P.I., Lochte F., Echeverria C., Pereira L.C., Coutinho J.T., Ferreira I.M., Novo C.M., Borges J.P. (2015). Thermal and magnetic properties of iron oxide colloids: Influence of surfactants. Nanotechnology.

[31] Huh Y.M., Jun Y.W., Song H.T., Kim S., Choi J.S., Lee J.H., Yoon S., Kim K.S., Shin J.S., Suh J.S. (2005). In vivo magnetic resonance detection of cancer by using multifunctional magnetic nanocrystals. J. Am. Chem. Soc..

[32] Suma P., Srinivas M., Sharada R., Nayanabhirama U., Satish R.B.S. (2015). PEGylation of superparamagnetic iron oxide nanoparticle for drug delivery applications with decreased toxicity: An in vivo study. J. Nanopart. Res..

[33] Liu G., Gao J., Ai H., Chen X. (2013). Applications and potential toxicity of magnetic iron oxide nanoparticles. Small.

[34] Ulbricht J., Jordan R., Luxenhofer R. (2014). On the biodegradability of polyethylene glycol, polypeptoids and poly(2-oxazoline)s. Biomaterials.

[35] Goy R.C., De Britto D., Assis O.B.G. (2009). A review of the antimicrobial activity of chitosan. Polimeros.

[36] Shukla S.K., Mishra A.K., Arotiba O.A., Mamba B.B. (2013). Chitosan-based nanomaterials: A state-of-the-art review. Int. J. Biol. Macromol..

[37] Javid A., Ahmadian S., Saboury A.A., Kalantar S.M., Rezaei-Zarchi S. (2013). Chitosan-coated superparamagnetic iron oxide nanoparticles for doxorubicin delivery: Synthesis and anticancer effect against human ovarian cancer cells. Chem. Biol. Drug Des..

[38] Bohara R.A., Thorat N.D., Pawar S.H. (2016). Role of functionalization: Strategies to explore potential nano-bio applications of magnetic nanoparticles. RSC Adv..

[39] Zhu N., Ji H., Yu P., Niu J., Farooq M.U., Akram M.W., Udego I.O., Li H., Niu X. (2018). Surface modification of magnetic iron oxide nanoparticles. Nanomaterials.

[40] Wu W., Wu Z., Yu T., Jiang C., Kim W.S. (2015). Recent progress on magnetic iron oxide nanoparticles: Synthesis, surface functional strategies and biomedical applications. Sci. Technol. Adv. Mater..

[41] Xu C., Sun S. (2013). New forms of superparamagnetic nanoparticles for biomedical applications. Adv. Drug Deliv. Rev..

[42] Rana A.-H., Sarah F.A.-A., Farah M., Maram S.K., Ahlam A.-B. (2020). Perceptive review on properties of iron oxide nanoparticles and their antimicrobial and anticancer activity. Sys. Rev. Pharm..

[43] Ivashchenko O., Lewandowski M., Peplińska B., Jarek M., Nowaczyk G., Wiesner M., Załęski K., Babutina T., Warowicka A., Jurga S. (2015). Synthesis and characterization of magnetite/silver/antibiotic nanocomposites for targeted antimicrobial therapy. Mater. Sci. Eng. C.

[44] Spiridis N., Socha R.P., Handke B., Haber J., Szczepanik M., Korecki J. (2011). Cluster–support interaction in Au-Fe_3_O_4_ system. Catal. Today.

[45] Zhang W., Rittmann B., Chen Y. (2011). Size effects on adsorption of hematite nanoparticles on E. coli cells. Environ. Sci. Technol..

[46] Cortajarena A.L., Ortega D., Ocampo S.M., Gonzalez-García A., Couleaud P., Miranda R., Belda-Iniesta C., Ayuso-Sacido A. (2014). Engineering Iron Oxide Nanoparticles for Clinical Settings. Nanobiomedicine.

[47] Qu C., Qian S., Chen L., Guan Y., Zheng L., Liu S., Chen W., Cai P., Huang Q. (2019). Size-dependent bacterial toxicity of hematite particles. Environ. Sci. Technol..

[48] Lee C., Kim J.Y., Lee W.I., Nelson K.L., Yoon J., Sedlak D.L. (2008). Bactericidal effect of zero-valent iron nanoparticles on Escherichia coli. Environ. Sci. Technol..

[49] Vega-Jiménez A.L., Vázquez-Olmos A.R., Acosta-Gío E., Álvarez-Pérez M.A., Seng Koh K., Loong Wong V. (2019). In vitro Antimicrobial Activity Evaluation of Metal Oxide Nanoparticles. Nanoemulsion-Properties, Fabrications and Applications.

[50] Samrot A.V., Sahithya C.S., Selvarani J., Purayil S.K., Ponnaiah P. (2021). A review on synthesis, characterization and potential biological applications of superparamagnetic iron oxide nanoparticles. Curr. Res. Green Sustain. Chem..

[51] Lee J.H., Ju J.E., Kim B.I., Pak P.J., Choi E.K., Lee H.S., Chung N. (2014). Rod-shaped iron oxide nanoparticles are more toxic than sphere-shaped nanoparticles to murine macrophage cells. Environ. Toxicol. Chem..

[52] Blanco E., Shen H., Ferrari M. (2015). Principles of nanoparticle design for overcoming biological barriers to drug delivery. Nat. Biotechnol..

[53] Godoy-Gallardo M., Eckhard U., Delgado L.M., de Roo Puente Y.J.D., Hoyos-Nogués M., Gil F.J., Perez R.A. (2021). Antibacterial approaches in tissue engineering using metal ions and nanoparticles: From mechanisms to applications. Bioact. Mater..

[54] Arakha M., Pal S., Samantarrai D., Panigrahi T.K., Mallick B.C., Pramanik K., Mallick B., Jha S. (2015). Antimicrobial activity of iron oxide nanoparticle upon modulation of nanoparticle-bacteria interface. Sci. Rep..

[55] Javanbakht T., Laurent S., Stanicki D., Wilkinson K.J. (2016). Relating the Surface Properties of Superparamagnetic Iron oxide nanoparticles (SPIONs) to their bactericidal effect towards a biofilm of streptococcus mutans. PLoS ONE.

[56] Polyak B., Friedman G. (2009). Magnetic targeting for site-specific drug delivery: Applications and clinical potential. Expert. Opin. Drug Deliv..

[57] Nowak-Jary J., Machnicka B. (2023). In vivo biodistribution and clearance of magnetic iron oxide nanoparticles for medical applications. Int. J. Nanomed..

[58] Polyak B., Friedman G. (2022). Pharmacokinetics of magnetic iron oxide nanoparticles for medical applications. J. Nanobiotechnol..

[59] Geng Y., Dalhaimer P., Cai S., Tsai R., Tewari M., Minko T., Discher D.E. (2007). Shape effects of filaments versus spherical particles in flow and drug delivery. Nat. Nanotechnol..

[60] Champion J.A., Mitragotri S. (2006). Role of target geometry in phagocytosis. Proc. Natl. Acad. Sci. USA.

[61] Park J.H., von Maltzahn G., Zhang L., Schwartz M.P., Ruoslahti E., Bhatia S.N., Sailor M.J. (2008). Magnetic Iron Oxide Nanoworms for Tumor Targeting and Imaging. Adv. Mater..

[62] Peng X.H., Qian X., Mao H., Wang A.Y., Chen Z.G., Nie S., Shin D.M. (2008). Targeted magnetic iron oxide nanoparticles for tumor imaging and therapy. Int. J. Nanomed..

[63] Sakulkhu U., Mahmoudi M., Maurizi L., Salaklang J., Hofmann H. (2014). Protein corona composition of superparamagnetic iron oxide nanoparticles with various physico-chemical properties and coatings. Sci. Rep..

[64] Chertok B., David A.E., Yang V.C. (2010). Polyethyleneimine-modified iron oxide nanoparticles for brain tumor drug delivery using magnetic targeting and intra-carotid administration. Biomaterials.

[65] Alexis F., Pridgen E., Molnar L.K., Farokhzad O.C. (2008). Factors affecting the clearance and biodistribution of polymeric nanoparticles. Mol. Pharm..

[66] Ge Y., Zhang Y., Xia J., Ma M., He S., Nie F., Gu N. (2009). Effect of surface charge and agglomerate degree of magnetic iron oxide nanoparticles on KB cellular uptake in vitro. Colloid Surf. B.

[67] Schweiger C., Hartmann R., Zhang F., Parak W.J., Kissel T.H., Rivera Gil P. (2012). Quantification of the internalization patterns of superparamagnetic iron oxide nanoparticles with opposite charge. J. Nanobiotechnol..

[68] Tran N., Mir A., Mallik D., Sinha A., Nayar S., Webster T.J. (2010). Bactericidal effect of iron oxide nanoparticles on Staphylococcus aureus. Int. J. Nanomed..

[69] Shebl R.I., Farouk F., Azzazy H.M.E.-S. (2017). Effect of surface charge and hydrophobicity modulation on the antibacterial and antibiofilm potential of magnetic iron nanoparticles. J. Nanomater..

[70] Zomorodian K., Veisi H., Mousavi S.M., Ataabadi M.S., Yazdanpanah S., Bagheri J., Mehr A.P., Hemmati S., Veisi H. (2018). Modified magnetic nanoparticles by PEG-400-immobilized Ag nanoparticles (Fe_3_O_4_@PEG-Ag) as a core/shell nanocomposite and evaluation of its antimicrobial activity. Int. J. Nanomed..

[71] Gong P., Li H., He X., Wang K., Hu J., Tan W., Zhang S., Yang X. (2007). Preparation and antibacterial activity of Fe_3_O_4_@Ag nanoparticles. Nanotechnology.

[72] Subbiahdoss G., Sharifi S., Grijpma D.W., Laurent S., van der Mei H.C., Mahmoudi M., Busscher H.J. (2012). Magnetic targeting of surface-modified superparamagnetic iron oxide nanoparticles yields antibacterial efficacy against biofilms of gentamicin-resistant staphylococci. Acta. Biomater..

[73] Ranmadugala D., Ebrahiminezhad A., Manley-Harris M., Ghasemi Y., Berenjian A. (2017). The effect of iron oxide nanoparticles on Bacillus subtilis biofilm, growth and viability. Process. Biochem..

[74] Azam A., Ahmed A.S., Oves M., Khan M.S., Habib S.S., Memic A. (2012). Antimicrobial activity of metal oxide nanoparticles against Gram-positive and Gram-negative bacteria: A comparative study. Int. J. Nanomed..

[75] Dash P., Raut S., Jena M., Nayak B. (2020). Harnessing the biomedical properties of ferromagnetic α-Fe2O3 NPs with a plausible formation mechanism. Ceram. Int..

[76] Ali M., Hamed M., Reza P. (2016). Design and development of a novel cellulose/γ-Fe_2_O_3_/Ag nanocomposite: A potential green catalyst and antibacterial agent. RSC Adv..

[77] Vasantharaj S., Sathiyavimal S., Senthilkumar P., LewisOscar F., Pugazhendhi A. (2019). Biosynthesis of iron oxide nanoparticles using leaf extract of Ruellia tuberosa: Antimicrobial properties and their applications in photocatalytic degradation. J. Photochem. Photobiol. B.

[78] Arami H., Khandhar A., Liggitt D., Krishnan K.M. (2015). In vivo delivery, pharmacokinetics, biodistribution and toxicity of iron oxide nanoparticles. Chem. Soc. Rev..

[79] Karlsson H.L., Cronholm P., Gustafsson J., Möller L. (2008). Copper oxide nanoparticles are highly toxic: A comparison between metal oxide nanoparticles and carbon nanotubes. Chem. Res. Toxicol..

[80] Ankamwar B., Lai T.C., Huang J.H., Liu R.S., Hsiao M., Chen C.H., Hwu Y.K. (2010). Biocompatibility of Fe(3)O(4) nanoparticles evaluated by in vitro cytotoxicity assays using normal, glia and breast cancer cells. Nanotechnology.

[81] McCormack P.L. (2012). Ferumoxytol: In iron deficiency anaemia in adults with chronic kidney disease. Drugs.

[82] Fidler J. (2007). MR imaging of the small bowel. Radiol. Clin. N. Am..

[83] Borcherding J., Baltrusaitis J., Chen H., Stebounova L., Wu C.M., Rubasinghege G., Mudunkotuwa I.A., Caraballo J.C., Zabner J., Grassian V.H. (2014). Iron oxide nanoparticles induce Pseudomonas aeruginosa growth, induce biofilm formation, and inhibit antimicrobial peptide function. Environ. Sci. Nano..

[84] Valdiglesias V., Fernández-Bertólez N., Kiliç G., Costa C., Costa S., Fraga S., Bessa M.J., Pásaro E., Teixeira J.P., Laffon B. (2016). Are iron oxide nanoparticles safe? current knowledge and future perspectives. J. Trace Elem. Med. Biol..

[85] Li W., Wei W., Wu X., Zhao Y., Dai H. (2020). The antibacterial and antibiofilm activities of mesoporous hollow Fe_3_O_4_ nanoparticles in an alternating magnetic field. Biomater. Sci..

[86] Bajpai I., Balani K., Basu B. (2014). Synergistic effect of static magnetic field and HA-Fe_3_O_4_ magnetic composites on viability of S. aureus and E. coli bacteria. J. Biomed. Mater. Res. B Appl. Biomater..

[87] Geilich B.M., Gelfat I., Sridhar S., van de Ven A.L., Webster T.J. (2017). Superparamagnetic iron oxide-encapsulating polymersome nanocarriers for biofilm eradication. Biomaterials.

[88] Wang X., Wu J., Li P., Wang L., Zhou J., Zhang G., Li X., Hu B., Xing X. (2018). Microenvironment-Responsive Magnetic nanocomposites based on silver nanoparticles/gentamicin for enhanced biofilm disruption by magnetic field. ACS Appl. Mater. Interfaces.

[89] Lakehal S., Douas H., Boukheris F. (2020). Antibacterial activity of α-Fe_2_O_3_ and α-Fe_2_O_3_@Ag nanoparticles prepared by urtica leaf extract. Nanotechnol. Russ..

[90] Vijayalakshmi K., Haq L., Noor U.L. (2021). Microwave-sonochemical synergistically assisted synthesis of hybrid Ni-Fe_3_O_4_/ZnO nanocomposite for enhanced antibacterial performance. Mater. Today Commun..

[91] Žalnėravičius R., Mikalauskaitė A., Niaura G., Paškevičius A., Jagminas A. (2019). Ultra-small methionine-capped Au^0^/Au^+^ nanoparticles as efficient drug against the antibiotic-resistant bacteria. Mater. Sci. Eng. C..

[92] Pan Y., Li J., Xia X., Wang J., Jiang Q., Yang J., Dou H., Liang H., Li K., Hou Y. (2022). β-glucan-coupled superparamagnetic iron oxide nanoparticles induce trained immunity to protect mice against sepsis. Theranostics.

[93] Shirazi M., Allafchian A., Salamati H. (2023). Design and fabrication of magnetic Fe_3_O_4_-QSM nanoparticles loaded with ciprofloxacin as a potential antibacterial agent. Int. J. Biol. Macromol..

[94] Rekha K., Nirmala M., Nair M.G., Anukaliani A. (2010). Structural, optical, photocatalytic and antibacterial activity of zinc oxide and manganese doped zinc oxide nanoparticles. Phys. B Condens. Matter.

[95] Tasnim N.T., Ferdous N., Rumon M.M.H., Shakil M.S. (2024). The promise of metal-doped iron oxide nanoparticles as antimicrobial agent. ACS Omega.

[96] Nguyen T.N.L., Do T.V., Nguyen T.V., Dao P.H., Trinh V.T., Mac V.P., Nguyen A.H., Dinh D.A., Nguyen T.A., Vo T.K.A. (2019). Antimicrobial activity of acrylic polyurethane/Fe_3_O_4_-Ag nanocomposite coating. Prog. Org. Coat..

[97] Kozenkova E., Levada K., Efremova M.V., Omelyanchik A., Nalench Y.A., Garanina A.S., Pshenichnikov S., Zhukov D.G., Lunov O., Lunova M. (2020). Multifunctional Fe_3_O_4_-Au nanoparticles for the MRI diagnosis and potential treatment of liver cancer. Nanomaterials.

[98] Ali A., Ovais M., Cui X., Rui Y., Chen C. (2020). Safety assessment of nanomaterials for antimicrobial applications. Chem. Res. Toxicol..

